# Supplementary material for: Ce-Doped Three-Dimensional Ni/Fe LDH Composite as a Sulfur Host for Lithium–Sulfur Batteries
Source: Nanomaterials (Basel). 2023 Aug 3;13(15):2244. doi: 10.3390/nano13152244 (PMC10421372; doi:10.3390/nano13152244)
Supplement: Supplementary file 1 [file nanomaterials-13-02244-s001.zip › nanomaterials-2504351-supplementary.pdf]

Ce-Doped Three-Dimensional Ni/Fe LDH Composite as a Sulfur Host for Lithium–Sulfur Batteries

Huiying Wei, Qicheng Li, Bo Jin * and Hui Liu

Key Laboratory of Automobile Materials, Ministry of Education, and School of Materials Science and Engineering, Jilin University, Changchun 130022, China; weihy22@mails.jlu.edu.cn (H.W.); liqc20@mails.jlu.edu.cn (Q.L.); liuhui20@mails.jlu.edu.cn (H.L.)

* Correspondence: jinbo@jlu.edu.cn

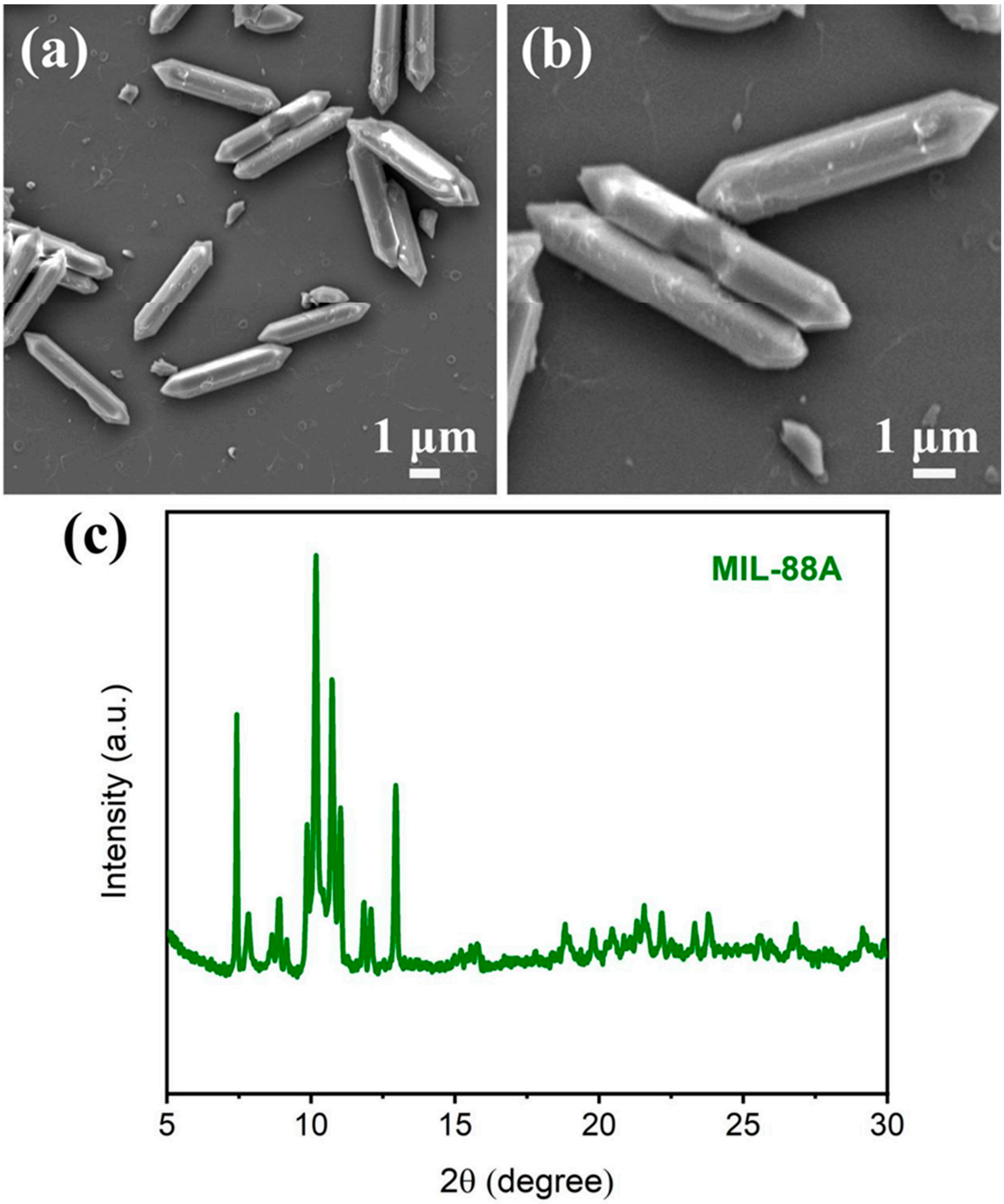

Figure S1. (a,b) FESEM images and (c) XRD pattern of MIL-88A precursor.

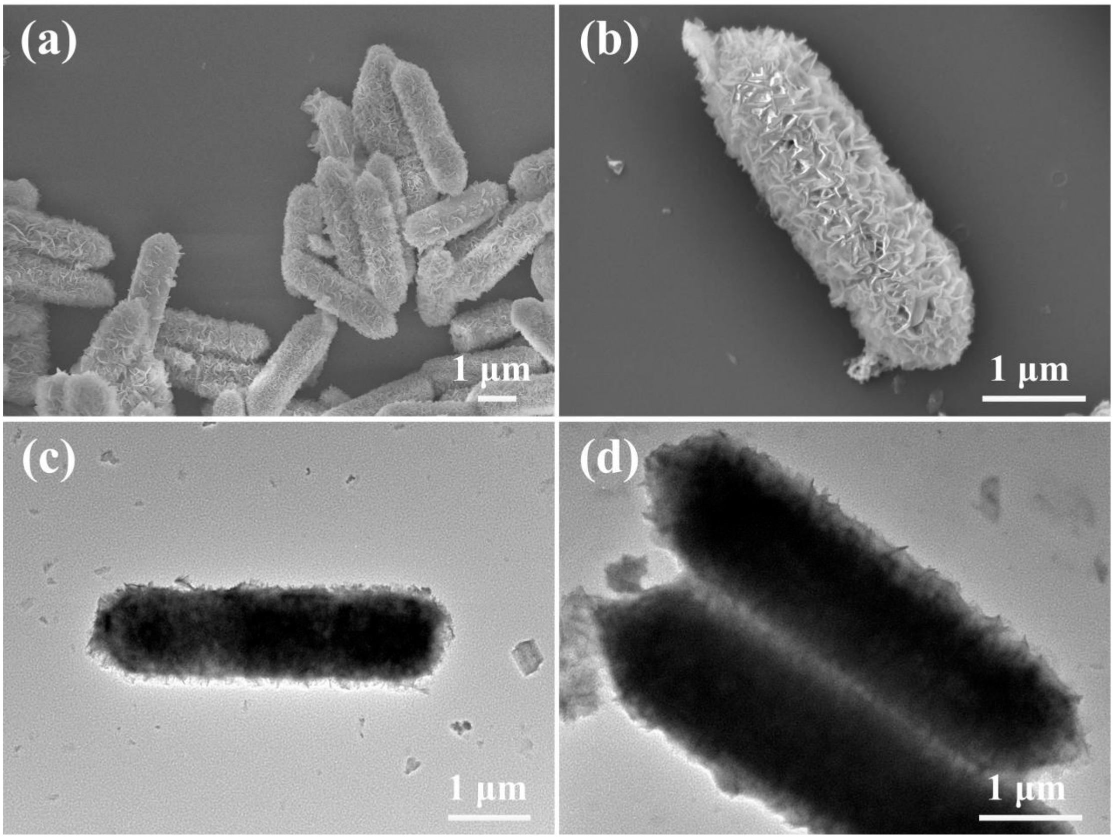

Figure S2. (a,b) FESEM images of Ce-Ni/Fe LDH. (c,d) TEM images of Ce-Ni/Fe LDH.

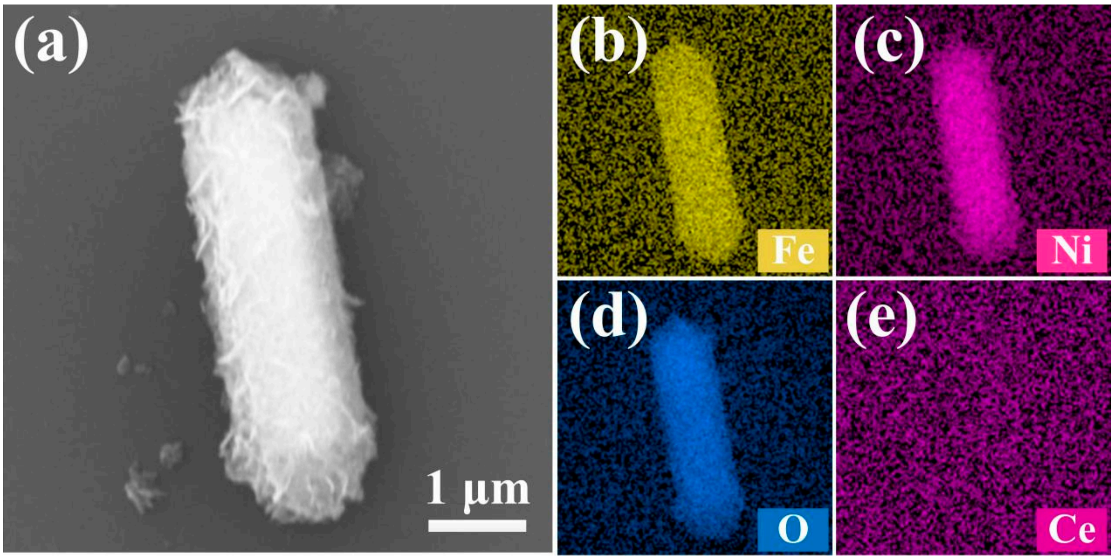

Figure S3. (a) SEM image and (b-e) corresponding elemental mappings of Ni/Fe LDH.

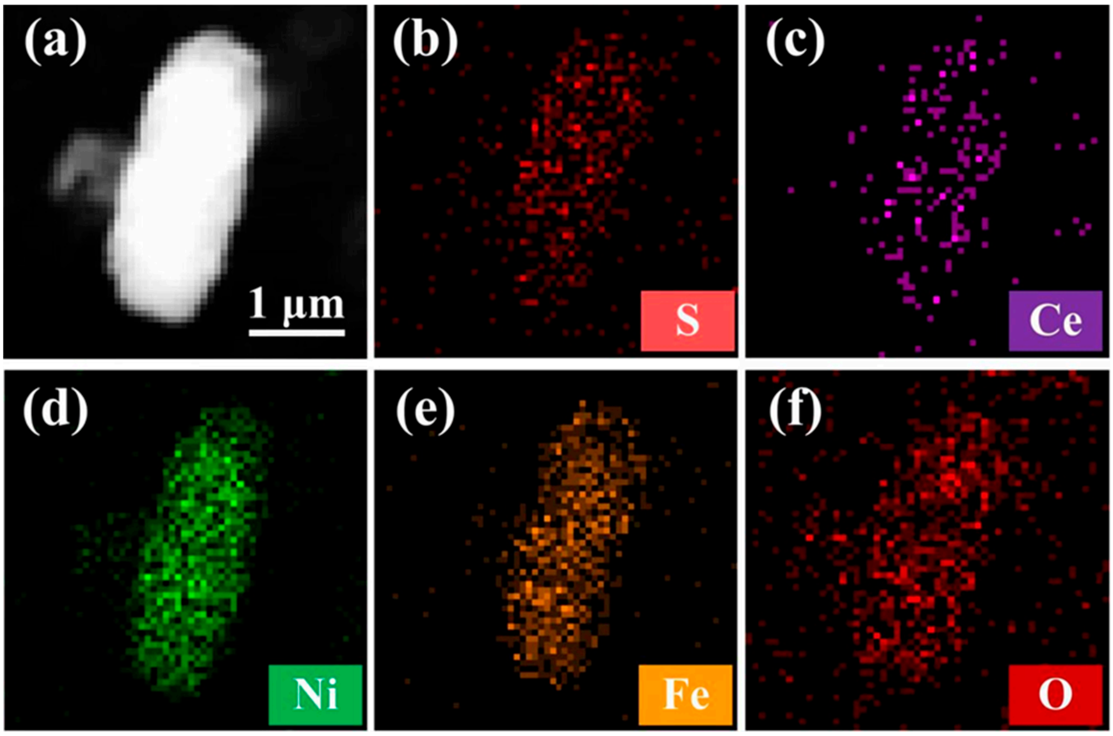

Figure S4. (a) STEM image and (b-f) corresponding elemental mappings of S@Ce-Ni/Fe LDH.

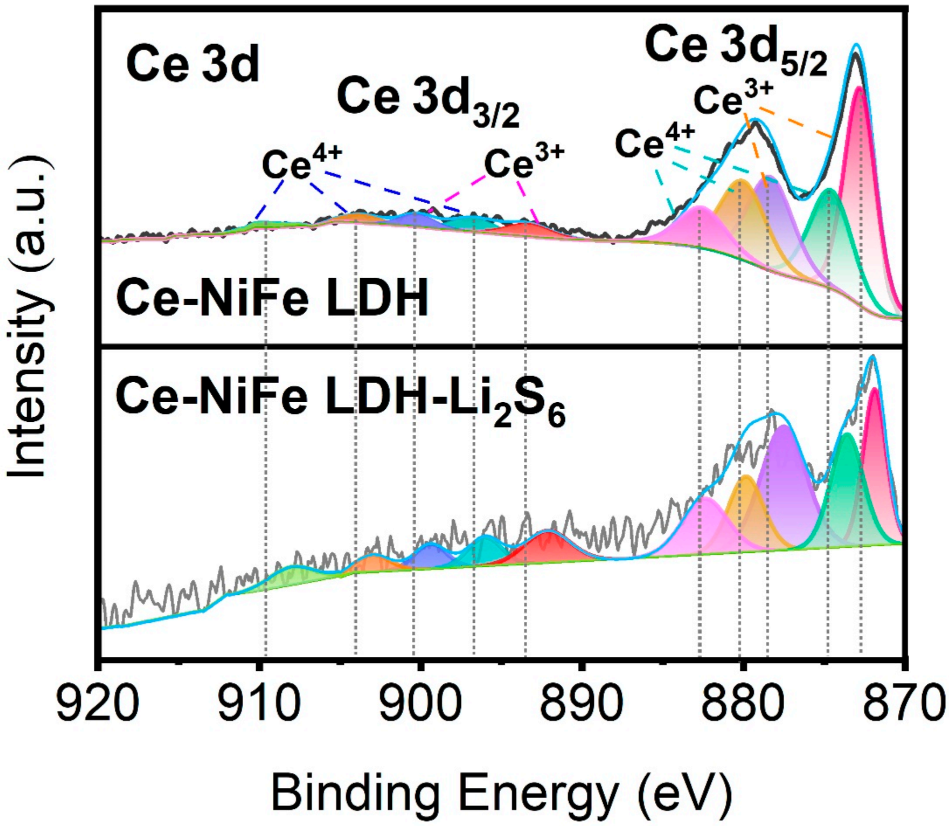

Figure S5. Ce 3d high-resolution XPS spectra of Ce-NiFe LDH and Ce-NiFe LDH-Li_2S_6.

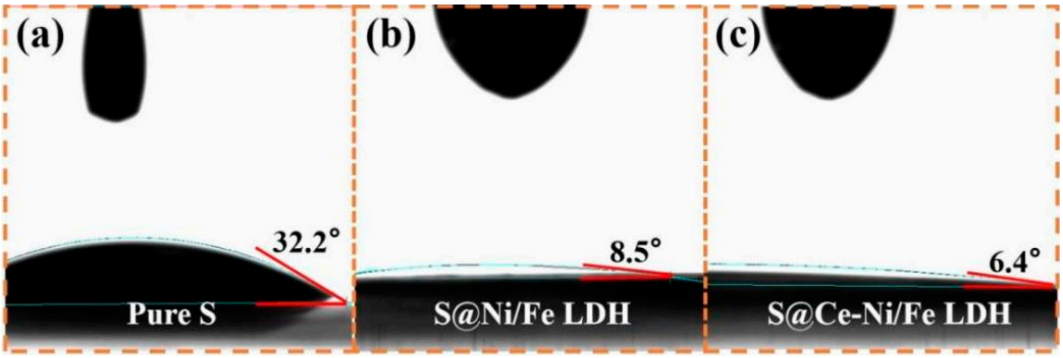

Figure S6. Contact angles between electrolyte and (a) pure S or (b) S@Ni/Fe LDH or (c) S@Ce-Ni/Fe LDH.

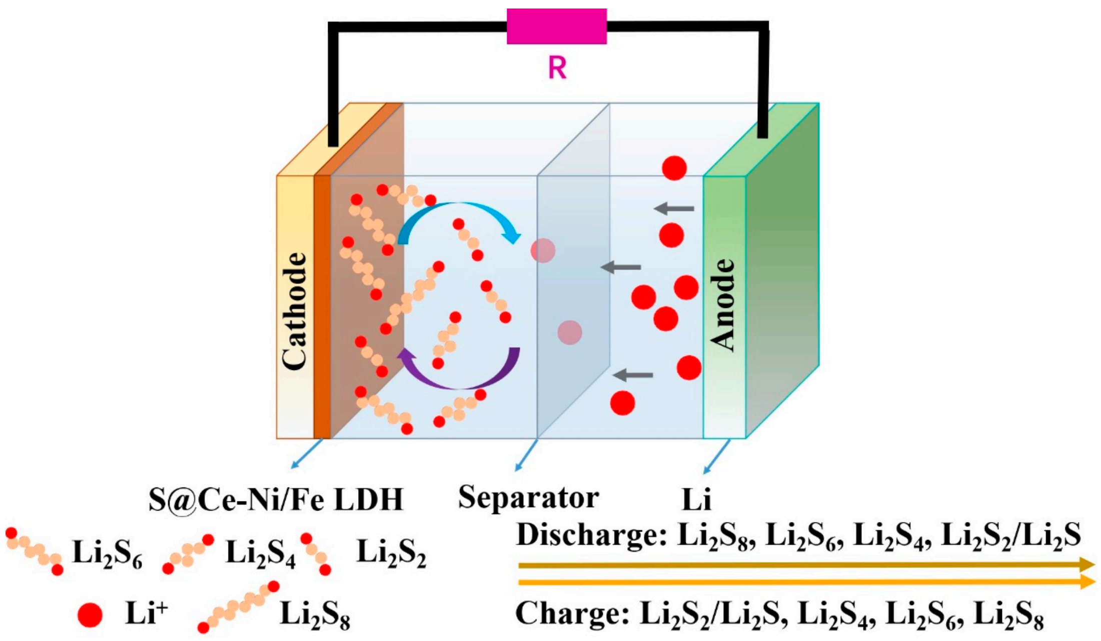

Figure S7. Schematic illustration of the redox reaction mechanism of lithium-sulfur battery with S@Ce-Ni/Fe LDH.

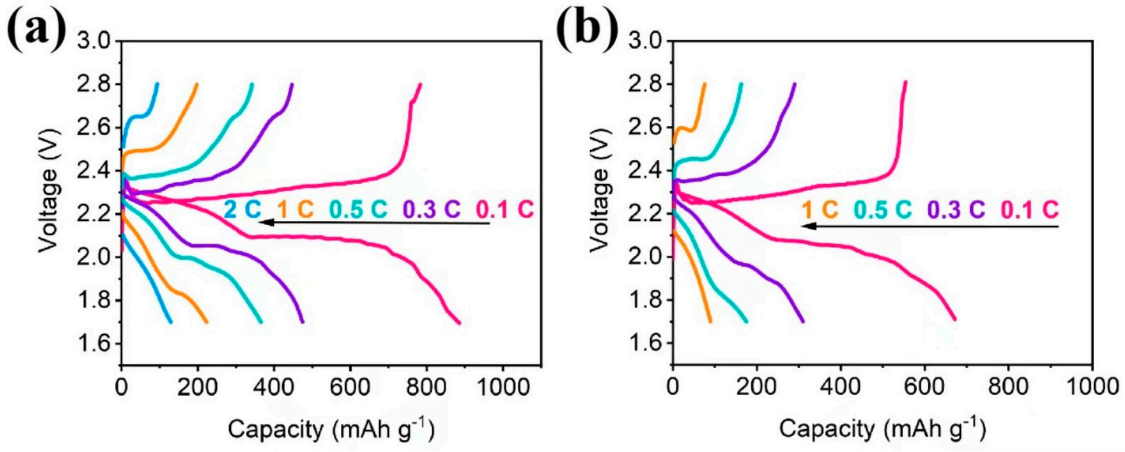

Figure S8. Charge/discharge curves of LSBs with (a) S@Ni/Fe LDH and (b) pure S at different current densities.

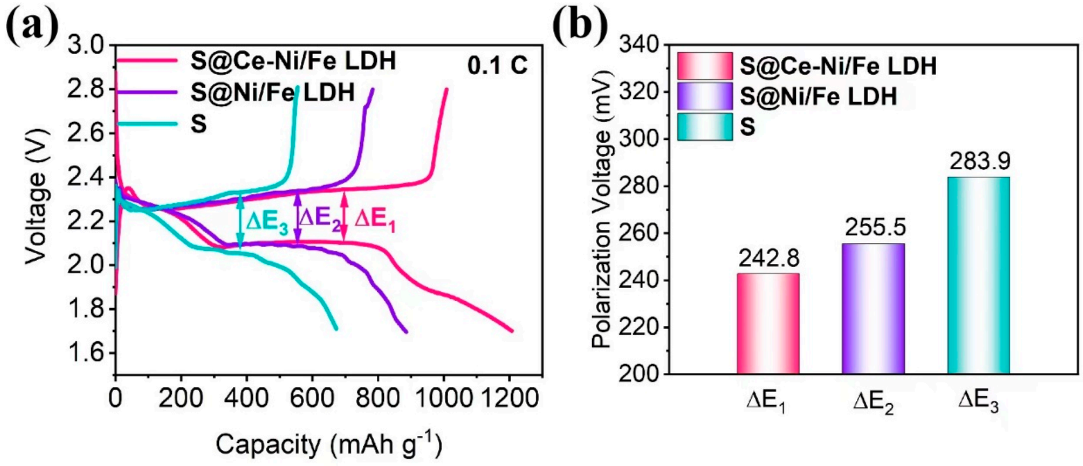

Figure S9. (a) The first charge/discharge curves and (b) polarization voltage histograms of LSBs with S@Ce-Ni/Fe LDH, S@Ni/Fe LDH, and pure S at 0.1 C.

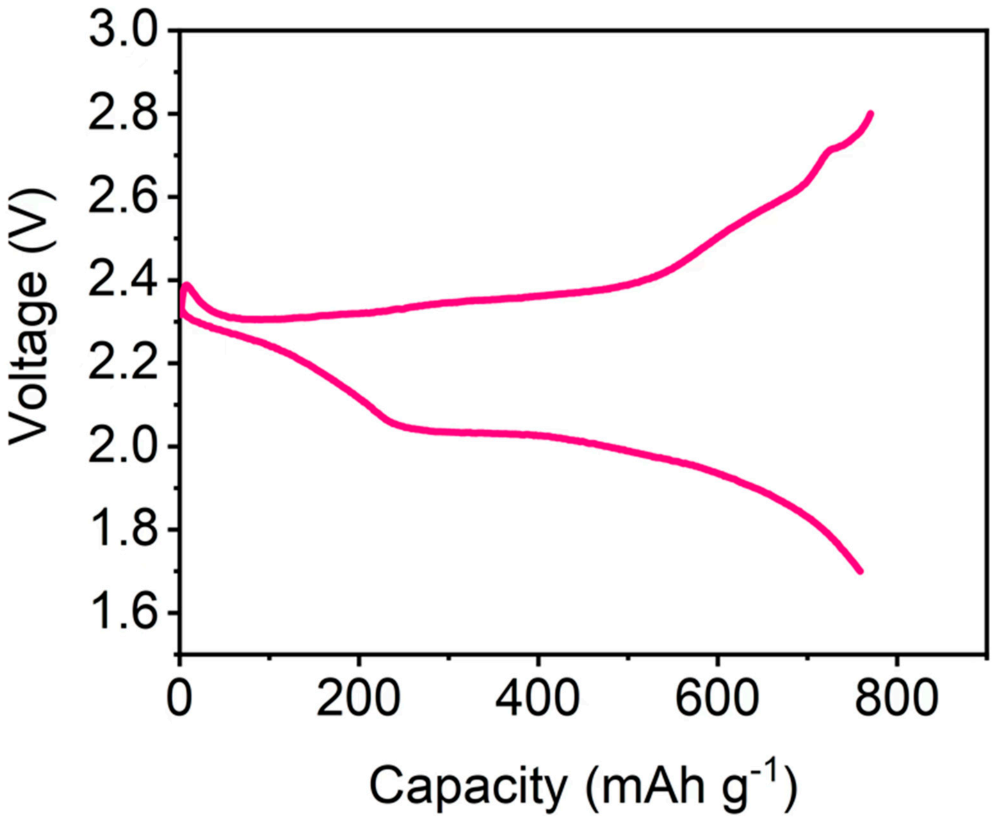

Figure S10. The 100th charge/discharge curves of lithium-sulfur battery with S@Ce-Ni/Fe LDH at 0.2 C.

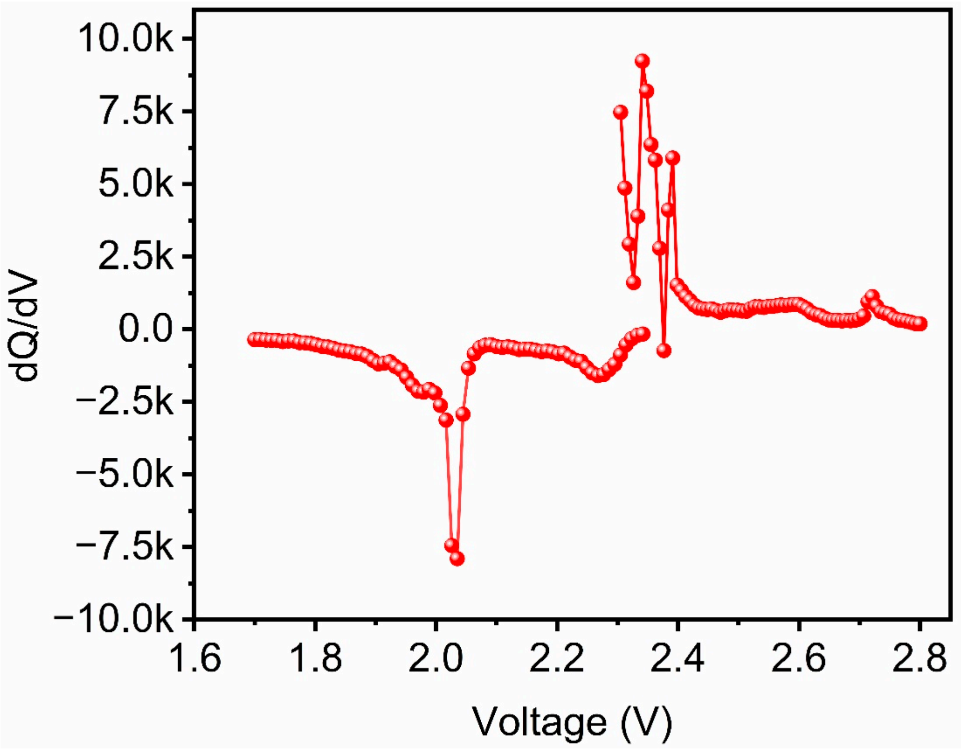

Figure S11. dQ/dV plot of lithium-sulfur battery with S@Ce-Ni/Fe LDH at 0.2 C during the 100th cycle.

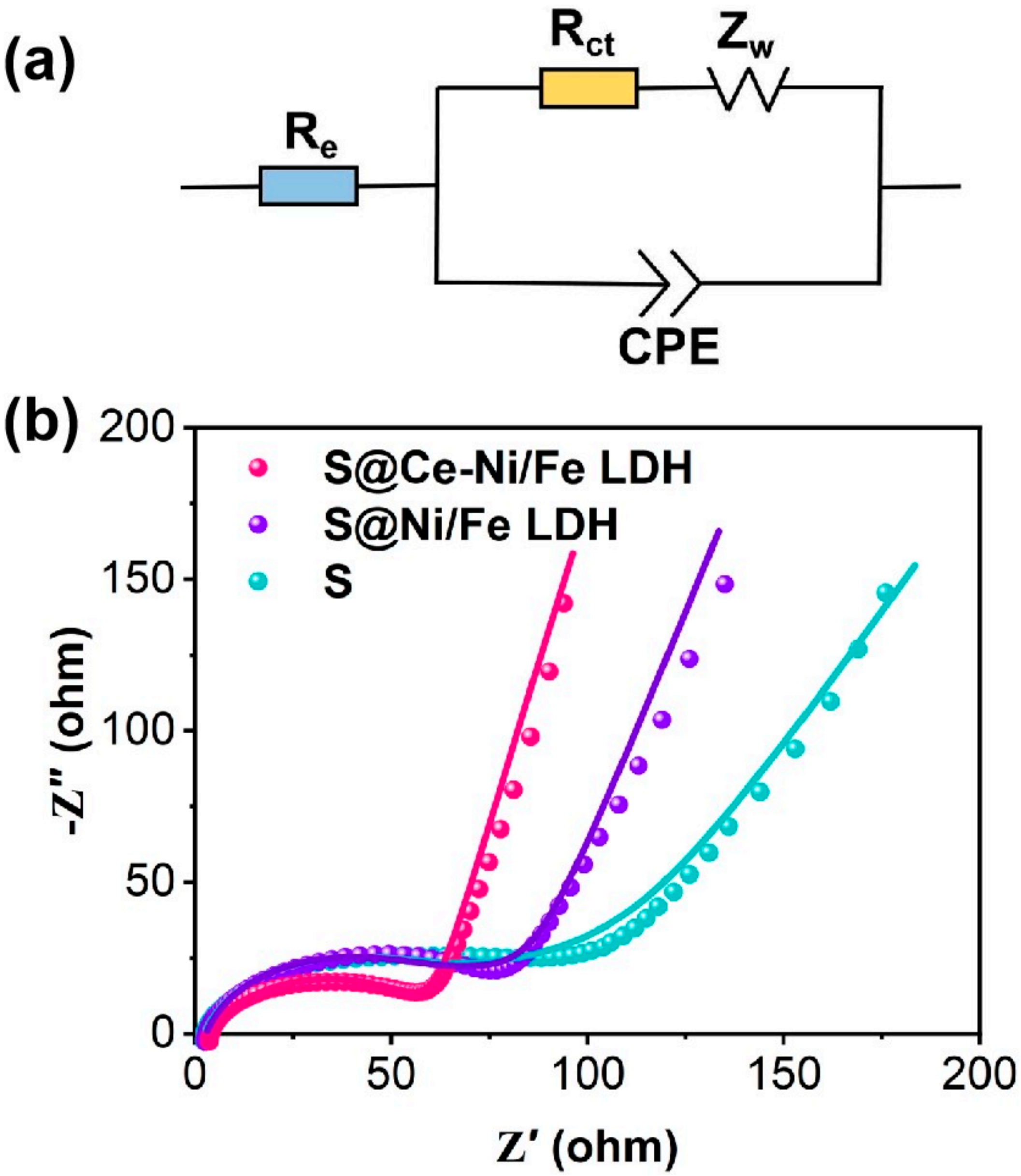

Figure S12. (a) The equivalent circuit diagram of LSBs with S@Ce-Ni/Fe LDH, S@Ni/Fe LDH, and pure S before cycling. (b) The fitting of EIS spectra of LSBs with S@Ce-Ni/Fe LDH, S@Ni/Fe LDH, and pure S before cycling.

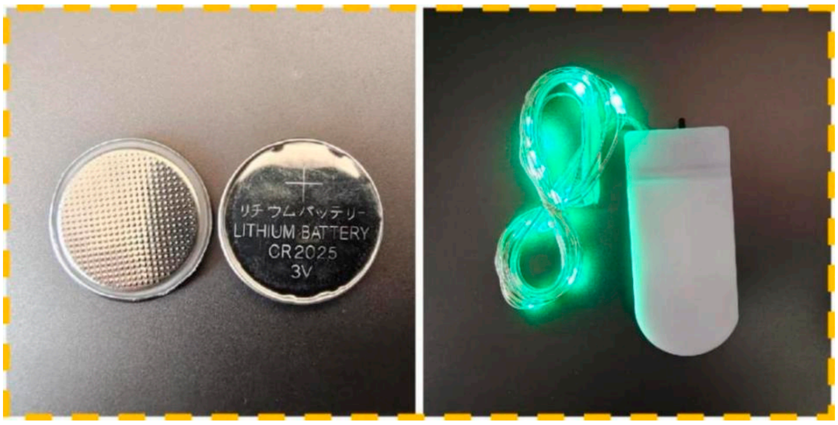

Figure S13. Digital photo of a light-emitting diode (LED) green lamp string lit up by two CR2025-type LSBs with S@Ce-Ni/Fe LDH connected in series.

Table S1. Comparison of lithium-sulfur batteries with other major battery types.

Cathode material	Theoretical capacity (mAh/g)	Work voltage (V)	Characteristic	Application area
**Sulfur**	**1675**	**2.1**	**Capacity decay**	**Lithium-sulfur batteries**
$LiFePO_4$	170	3.2	Capacity decay	Lithium-ion batteries
$LiCoO_2$	274	3.7	Good cycle performance	Lithium-ion batteries

Table S2. Comparison of electrochemical performance of lithium-sulfur battery with S@Ce-Ni/Fe LDH with previously reported literatures.

Sample	Current density	Cycle number	Discharge capacity (mAh g^{-1})	Capacity decay rate per cycle (%)	Ref.
CH@LDH	0.5 C	100	491	0.342	17
LDH/rGO	0.2 C	100	573	0.404	23
Co-Fe LDH	0.5 C	200	557	-	52
NiFe-LDH	0.2 C	100	503	0.256	53
**Ce-Ni/Fe LDH**	**0.2 C**	**100**	**759**	**0.28**	**This work**
	**1 C**	**1000**	**308**	**0.067**	

References

17 Zhang, J.-T.; Hu, H.; Li, Z.; Lou, X.-W. Double-Shelled Nanocages with Cobalt Hydroxide Inner Shell and Layered Double Hydroxides Outer Shell as High-Efficiency Polysulfide Mediator for Lithium-Sulfur Batteries. *Angew. Chem. Int. Edit.* **2016**, 55, 3982-3986.

23 Xu, F.-C.; Dong, C.-W.; Jin, B.; Li, H.; Wen, Z.; Jiang, Q. MOF-Derived LDH Wrapped with rGO as an Efficient Sulfur Host for Lithium-Sulfur Batteries. *J. Electroanal. Chem.* **2020**, 876, 114545.

51 Wei, H.-J; Liu, J.; Liu, Y.; Wang, L.; Li, L.-L.; Wang, F.; Ren, X.-Y.; Ren, F.-Z. Hollow Co-Fe LDH as an Effective Adsorption/Catalytic Bifunctional Sulfur Host For High-Performance Lithium-Sulfur Batteries. *Compos. Commun.* **2021**, 28, 100973.

52 Liu, Q.; Zhang, Y.-Q.; Zhou, Y.-B.; Wang, M.; Li, R.-L.; Yue, W.-B. Layered Bouble Hydroxides Used as the Sulfur Hosts for Lithium-Sulfur Batteries and the Influence of Metal Composition on Their Performance. *J. Solid State Electr.* **2023**, 27, 797-807.
